# Genome Sequence of the K139-Like Phage VcP032 Originating from *Vibrio cholerae* O1 El Tor Ogawa Serotype

**DOI:** 10.1128/genomeA.00492-16

**Published:** 2016-07-21

**Authors:** Claudia Jäckel, Eckhard Strauch, Jens Andre Hammerl

**Affiliations:** a Unit Molecular Diagnostics and Genetics, Department of Biological Safety, Federal Institute for Risk Assessment, Berlin, Germany; b Unit Product Hygiene and Disinfection Strategies, Department of Biological Safety, Federal Institute for Risk Assessment, Berlin, Germany; c Unit Epidemiology, Zoonoses and Antimicrobial Resistance, Department of Biological Safety, Federal Institute for Risk Assessment, Berlin, Germany

## Abstract

*Vibrio cholerae* is the cause of large cholera outbreaks, especially in endemic regions with high poverty and inadequate sanitation. Here, we announce the complete genome sequence of the virulence-associated broad host range *V. cholerae* phage VcP032, including a brief summary of its genotypic and phenotypic features.

## GENOME ANNOUNCEMENT

*Vibrio cholerae* strains of serogroup O1 or O139 are highly infectious bacteria ubiquitously present in coastal, river, and estuarine ecosystems of endemic regions. Seven cholera pandemics were recorded in the past two centuries. A total of 1.4 to 4.3 million human cases caused by the O1 El Tor serotype were estimated for the prevailing pandemic, originating in Indonesia in 1967 (1, 2). Human infections are often asymptomatic or associated with mild or painless, watery diarrhea. However, without medication, the human lethality rate can reach up to 60% (3).

Bacteriophages have a high impact on the pathogenicity of *V. cholerae*, as some virulence determinants are encoded by temperate phages (4). The cholera toxin is encoded by a filamentous phage that is present in toxigenic stains of environmental and clinical origin (5). Another virulence factor (*glo*) is encoded by myoviruses of the K139-phage family, which are also widely distributed among *V. cholerae* strains (6).

Here, we announce the phage genome of VcP032, recovered from *V. cholerae* serotype O1 El Tor Ogawa strain CH32, which was isolated in 1973. Phage induction was activated by mitomycin C (12.5 µg/ml) treatment (7). Concentrated phages were purified by CsCl-step gradient centrifugation (8). VcP032 revealed a K139-like myoviridal morphology and strong lytic activity (turbid plaques of 0.5 to 1 mm in size) on eight out of 38 tested *V. cholerae* O1 strains. Further tests with *V. cholerae* serotypes O139 (*n* = 4) and non-O1/O139 (*n* = 145), as well as strains of the species *V. parahaemolyticus* (*n* = 20) and *V. vulnificus* (*n* = 50), showed no plaque formation. Next-generation sequencing was performed to determine the content of stress-released phages and their impact on the virulence and evolution of *V. cholerae* isolates showing high human infection rates during local outbreaks. Therefore, VcP032 DNA was extracted by incubating CsCl-purified phages (~10^9^ PFU) with proteinase K/SDS solution (10 mM Tris-HCl [pH 7.5], 1 mM EDTA, 0.5% SDS supplemented with 40 mg/ml proteinase K) at 56°C for 2 h, followed by phenol-chloroform treatment and ethanol precipitation (9). Whole-genome sequencing was performed with Illumina HiSeq (run type: paired-end reads; read length: 2 × 100 bp) by GATC Biotech AG (Konstanz, Germany). A *de novo* genome assembly based on 13,255,917 reads was developed using Newbler version 2.8 software (454 Life Sciences, Branford, CT, USA), yielding a single linear contig with a sequence coverage of >500-fold per consensus base. The VcP032 sequence contains 33,108 bp with an average G+C content of 46.1%. Gene prediction and annotation of the phage genome was carried out using MyRAST (10–12). A total of 46 coding sequences and 15 transcription terminators (13) were identified. Genome comparison (14) indicates that VcP032 is closely related to *Vibrio* phages K139 (99%, AF125163.2) (6), kappa (99%, AB374228.1), and VPUSM8 (99%, KF361475.1), as well as to a cryptic prophage of the *V. cholerae* genome MJ-1236 (99%, CP001485.1). Differences between VcP032 and its relatives are mainly based on sequence deviations (i.e., single nucleotide polymorphisms, deletions). Further experimental analyses are necessary to predict the effect of sequence alterations on VcP032 properties. As VcP032 harbors a gene for a Glo-like periplasmic protein homolog (VcP032_03), this phage may increase the virulence of lysogenic *V. cholerae* strains (15).

### Nucleotide sequence accession number.

The genome sequence of VcP032 has been deposited in GenBank under the accession number KX058879.
